# A Review of Eight High-Priority, Economically Important Viral Pathogens of Poultry within the Caribbean Region

**DOI:** 10.3390/vetsci5010014

**Published:** 2018-01-26

**Authors:** Arianne Brown Jordan, Victor Gongora, Dane Hartley, Christopher Oura

**Affiliations:** 1 Department of Basic Veterinary Sciences, School of Veterinary Medicine, The University of the West Indies (St. Augustine), Eric Williams Medical Sciences Complex, Mount Hope, Trinidad and Tobago; brown.arianne@gmail.com; 2 Belize Poultry Association, Belmopan, Belize; bz.victor@gmail.com; 3 Veterinary Services Laboratory, Guyana Livestock Development Authority, Agriculture Road, Mon Repos, East Coast Demerara; danehart2002@yahoo.com

**Keywords:** Caribbean, avian influenza virus, infectious bronchitis virus, Newcastle disease virus, infectious laryngotracheitis virus, avian metapneumovirus, infectious bursal disease, fowl adenovirus group 1, egg drop syndrome virus

## Abstract

Viral pathogens cause devastating economic losses in poultry industries worldwide. The Caribbean region, which boasts some of the highest rates of poultry consumption in the world, is no exception. This review summarizes evidence for the circulation and spread of eight high-priority, economically important poultry viruses across the Caribbean region. Avian influenza virus (AIV), infectious bronchitis virus (IBV), Newcastle disease virus (NDV), infectious laryngotracheitis virus (ILTV), avian metapneumovirus (aMPV), infectious bursal disease virus (IBDV), fowl adenovirus group 1 (FADV Gp1), and egg drop syndrome virus (EDSV) were selected for review. This review of serological, molecular, and phylogenetic studies across Caribbean countries reveals evidence for sporadic outbreaks of respiratory disease caused by notifiable viral pathogens (AIV, IBV, NDV, and ILTV), as well as outbreaks of diseases caused by immunosuppressive viral pathogens (IBDV and FADV Gp1). This review highlights the need to strengthen current levels of surveillance and reporting for poultry diseases in domestic and wild bird populations across the Caribbean, as well as the need to strengthen the diagnostic capacity and capability of Caribbean national veterinary diagnostic laboratories.

## 1. Introduction

Viral pathogens cause devastating economic losses in poultry industries worldwide. The Caribbean region has a thriving and growing poultry industry, which frequently suffers from the economic effects of disease. The Caribbean poultry industry is of high importance to the region, not only in relation to the production of locally consumed food, but also for the production of poultry products for trade and export. The production and trade of poultry and poultry products are substantial contributors to gross domestic product (GDP) for many Caribbean countries [1]. In the Latin American and Caribbean region, which ranks second in the world for poultry consumption, Caribbean countries have some of the highest rates of poultry consumption per capita [1]. Data collected in 2011 ranked poultry meat consumption highest in Saint Vincent and the Grenadines, Saint Lucia, Antigua and Barbuda, Saint Kitts and Nevis, Bahamas, and Trinidad and Tobago (T & T), with rates well above that of other countries in the Americas [1]. A report published in 2003 on livestock production in Latin America and the Caribbean predicted that 18.2 million tons of poultry would be produced by the region in 2015 [2]. This figure actually reached 23.8 million tons by 2014 [3]. Aside from meat production, egg yield and egg consumption continues to grow in the Latin American and Caribbean regions, despite frequent disease outbreaks and rising production costs [4].

Historically, surveillance and reporting has been lacking in the Caribbean. However, improved knowledge related to the circulation and spread of viral pathogens affecting poultry within the Caribbean is critical to enable both public health and veterinary health sectors to introduce more effective prevention and control strategies. This would have a direct effect on improving the health and welfare of poultry, as well as increasing the profits for poultry farmers, across the Caribbean region.

In this comprehensive review, studies reporting previous and current detections of eight high-priority viral pathogens of poultry within the Caribbean have been examined. For the sake of this review, all Caribbean island states have been included (Anguilla, Antigua and Barbuda, Aruba, Bahamas, Barbados, British Virgin Islands, Caribbean Netherlands, Cayman Islands, Cuba, Curaçao, Dominica, Dominican Republic, Grenada, Guadeloupe, Haiti, Jamaica, Martinique, Montserrat, Puerto Rico, Saint Barthélemy, St. Kitts and Nevis, St. Lucia, St. Martin/St. Maarten, St. Vincent and the Grenadines, T & T, Turks and Caicos Islands, Virgin Islands), as well as the Caribbean Community (CARICOM) mainland member states: Belize, Guyana, and Suriname. Selection of the eight viral pathogens for review was based on their global and regional importance and include avian influenza virus (AIV), infectious bronchitis virus (IBV), Newcastle disease virus (NDV), infectious laryngotracheitis virus (ILTV), avian metapneumovirus (aMPV), infectious bursal disease virus (IBDV), fowl adenovirus group 1 (FADV Gp1), and egg drop syndrome virus (EDSV).

This review aims to collate and summarize the information and data available on the occurrence of eight priority viruses of poultry within the Caribbean region. The review provides important information related to the presence/absence of these viruses in Caribbean countries and, importantly, highlights knowledge gaps from a pathogen and a country perspective for targeting surveillance activities in the region. It should be noted that, although outbreak events can inform on the historic exposure of a population to disease, it cannot inform on the current epidemiological situation in the countries. Furthermore, because the surveillance and reporting practices may vary between pathogens, across countries and over time, a review of the published literature cannot provide a reliable comparison of the epidemiological situation across pathogens or countries.

**Figure 1:**
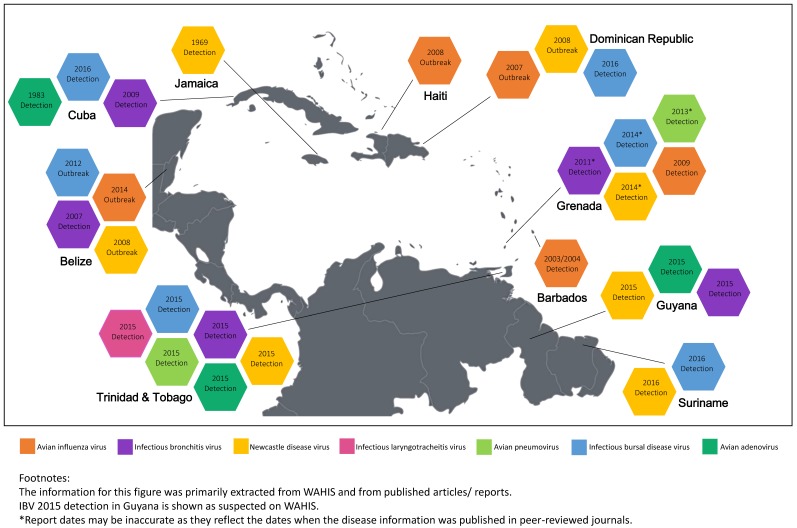
Identification of high-priority poultry virus across the Caribbean region.

For the purpose of this review, reports and articles were searched for in scholarly literature publication databases (Google Scholar, PubMed, and CAB Direct). Search terms used were specific country names, combined with the full names and acronyms for the eight viruses. Additionally, the key words “avian,” “poultry,” “Caribbean,” and “surveillance” were also used. Information related to the notification and monitoring of disease status information was obtained from the World Organisation for Animal Health (OIE) platform WAHIS (World Animal Health Information System). In some instances, information was also sourced from and confirmed by relevant poultry and veterinary officers in the various countries. Searches were conducted from the earliest available date in the database up to October 2017, and any reports thereafter were not presented in the review. Relevant reports and publications related to the eight prioritized viruses in any Caribbean country were considered for inclusion in the review. All sources were assessed by the authors for relevance, credibility, and scientific strength to ensure the thoroughness and accuracy of the review. The information related to the identification of the eight priority viruses within the Caribbean region is summarized in Figure 1.

### Poultry Vaccination Practices in the Caribbean Region

Worldwide, the use of vaccines is seen as critical for the prevention and control of many economically damaging outbreaks of poultry diseases. In Caribbean countries, the use of vaccination to prevent poultry diseases is variable and is mainly influenced by the size of the poultry industry in the various countries. Many of the larger poultry-producing CARICOM countries (for example, T & T, Jamaica, Belize, Barbados, and Guyana) with large poultry (broiler and layer) operations have structured and rigid regimes of vaccination in place, while smaller island states (for example, Grenada and St. Lucia), with smaller poultry and egg production operations, often do not vaccinate their poultry. The larger intensive broiler and layer production units in the Caribbean routinely vaccinate their birds against IBV, NDV, and IBDV, whereas some smaller semi-intensive and backyard operations often do not carry out vaccination against these three viral pathogens. Routine vaccination is not carried out in the region against other viral pathogens (AIV, ILTV, APV, FADV Gp1, and EDSV) included in this review, although occasional vaccination against FADV Gp1 has been carried out in the face of disease outbreaks. All reports/publications describing the detection of viruses and outbreaks of disease in both vaccinated and unvaccinated poultry were reviewed. When information pertaining to vaccination history was given in the relevant report/publication, this information was included.

## 2. Avian Influenza Virus

AIV is a member of the Orthomyxoviridae family and is known to cause infection in many susceptible bird species [5]. The virus can cause severe infection with high mortality rates, depending on the pathogenicity of the infecting strain [6]. Due to its segmented RNA genome, AIV is prone to reassortment, sometimes resulting in the emergence of zoonotic viruses [7]. AIV has a wild bird reservoir and this feature is often implicated in the spread of infection to poultry dwellings [8,9]. Outbreaks of avian influenza (AI) have occurred in a few countries across the Americas in recent years. Many studies have reported the isolation and molecular characterization of AIV strains across the region, but most outbreak incidents have been isolated to individual countries, seldom crossing borders [10,11]. Different strains of AIV, including highly pathogenic strains, have caused extensive outbreaks of disease, resulting in severe economic losses in some countries in the Americas. Most notable, recent outbreaks in Mexico and the USA have been extremely damaging to local economies. The recent 2015 outbreak in the USA resulted in direct losses of USD $1.6 billion and a ripple effect worldwide, including in the Caribbean, due to trade restrictions resulting from the outbreak [12]. Multiple outbreaks of LPAI H5N2 occurred in the US throughout 2015 in commercial chicken and turkey flocks, and there were nine reports of H5N2 in wild birds in the USA in 2015. The rapid spread and high levels of circulation of the H5N2 strain observed in the US during this period highlights the risk of transmission to the Caribbean territories through the importation of day-old chicks and the migration of wild birds. The outbreak of highly pathogenic H7N3 in Mexico in 2012 resulted in direct losses of USD $504 million. Mexico is still experiencing occasional outbreaks of disease caused by this virus strain, adding to the overall cost burden [13]. Mexico shares a border with Belize, so the risk of spread of AIV from Mexico to Belize is high. No emergency or preventative vaccination has previously been carried out against AIV in any of the reviewed Caribbean states, although some neighboring nations, such as Mexico, has carried out preventative and emergency vaccination for AIV in the past. Although vaccines against AIV can control and prevent disease, their use in the Caribbean to prevent AIV infections is not justifiable, given the lack of AIV outbreaks in the region. In the event of previous outbreaks in the region, rapid depopulation of affected farms, combined with heightened levels of sanitation and biosecurity, have been used as the methods of choice for the control and eradication of AIV.

In the CARICOM mainland member states, an outbreak of low pathogenic AI (H5N2) was reported in Belize in December 2014 in a broiler breeder flock [13]. The AIV from Belize was a North American LPAI H5N2 virus, which showed 98.8% similarity at a nucleotide level to an H5N2 (A/CK/Mexico/2012 H5N2) strain isolated from Mexico in 2012 [14]. At a high cost for control and eradication, Belize regained free status for AI in accordance with the OIE Terrestrial Animal Health Code (Article 10.4.3) in September 2015. There have been no previous reports of AI in poultry from the other two mainland states, Suriname and Guyana. The first reports of AIV in Caribbean island states was in 2007, when a low pathogenic (LP) H5N2 virus was detected on the island of Hispaniola, in the Dominican Republic (DR), in December 2007 and subsequently in Haiti in May 2008 [15]. The LPAI virus from the DR showed 95.5% similarity to a strain that had been circulating in the USA since 1994. Ten years later, in 2017, a different lineage of LP H5N2 serotype was again reported to be present in the DR [16]. These reports demonstrated the likely ability of the virus to spread from the North American continent to Caribbean island states [11]. A study carried out in Barbados isolated two H4N3 AIV strains from wild migratory birds, highlighting the risk of transmission of AIV strains from wild birds to local poultry flocks and the potential for regional spread of AIV through migration pathways [17]. A 2009 serological study carried out in Grenada found that 18.8% of backyard poultry had antibodies to AIV; however, in a subsequent study, no viral antibodies were detected in commercial layers and broilers on the island [18,19]. In T & T, a sero-surveillance study carried out on layer population on both islands of Trinidad and Tobago revealed no antibodies to AIV in the tested population [20].

Although there have been only a limited number of reports of AIV from Caribbean island states, the potential always exists for AIV to emerge and circulate on these islands. The aquatic wild bird reservoir, the migration patterns of wild birds [8,21], the proximity of the island states to the American continents, and both legal and illegal trade contribute to the risk of introduction. The Caribbean region is subject to the migration of thousands of wintering birds yearly from North America, where frequent cases of AIV occur [22]. This emphasizes the need for preparedness and vigilance across the Caribbean region, especially during the wild bird migratory season, and the need for both active and passive surveillance in domestic and wild birds. It should also be noted that many veterinary diagnostic laboratories in Caribbean countries do not currently have the local laboratory capacity to diagnose AIV in their countries, making it necessary for them to send samples from suspected outbreaks to OIE reference laboratories for confirmation. Caribbean laboratories should be supported to build diagnostic capacity, so they are capable of carrying out serological and molecular testing as part of AI surveillance programs, and in response to viral incursions.

## 3. Infectious Bronchitis Virus

IBV is a member of the Coronaviridae family that causes a highly infectious respiratory disease in poultry [23]. Coronaviruses are highly variable RNA viruses, which can result in the continuous evolution of new strains of the virus with different degrees of pathogenicity [24]. Some countries across the Caribbean with large poultry industries (e.g., T & T, Belize, and Jamaica) carry out routine vaccination against IBV to prevent significant economic losses from outbreaks of clinical disease. There is regional evidence, however, that the field circulating viruses (different serotypes and variants) are in some cases evading vaccination as clinical signs of disease have been observed in vaccinated flocks [25,26]. Of the mainland CARICOM countries, Belize and Guyana have reported the presence of IBV in their poultry [13]. Unfortunately, details of the nature of the circulating strains, the extent of circulation, and the clinical and economic consequences are lacking.

Of the Caribbean island states, Cuba has reported antibodies against IBV in poultry showing clinical signs of disease and have carried out molecular and phylogenetic analysis of the circulating viruses. Novel field strains of IBV were identified to be circulating and causing disease in vaccinated populations of birds that were genetically different to the IBV strains used in the live vaccine [25,27]. This observation of vaccine failure, combined with high levels of genetic divergence between field and vaccine strains, led the authors to recommend that Cuba fully review its IBV vaccination programs. Another study in Cuba revealed rising antibody titers independent of additional vaccine doses in layer birds, indicating that field strains of IBV were circulating in the layer birds [28]. A serological study carried out in T & T found that 100% of the sampled unvaccinated layer birds on both islands were antibody-positive for IBV [20]. A study published in 2011 from Grenada reported a 31% detection rate of antibodies to IBV in a variety of avian species, including broilers, free range chicken, ducks, and turkeys [29]. When the results were subdivided, 77.5% of the positive samples were found to be from free range chickens, while 21% were from commercial broilers. No vaccination against IBV is carried out in Grenada. The circulating IBV viruses were not identified and characterized in the studies from Grenada and T & T, so it is currently unknown whether field or vaccine strains of IBV are circulating on these islands.

## 4. Newcastle Disease Virus

NDV is a member of the Paramyxoviridae family that affects both poultry and wild birds. The disease can range in severity depending on the infecting virus strain [30]. Newcastle disease (ND) is considered endemic in many countries worldwide and has been identified as a major cause of disease in poultry as far back as the early 19th century [31], particularly in backyard type farms [32]. Countries with large poultry industries generally vaccinate their intensively produced layer and broiler poultry against NDV, but backyard flocks are often left unvaccinated.

In the CARICOM mainland member states, outbreaks of ND have never been reported from Guyana, where little is known about the occurrence NDV in commercial or backyard poultry flocks. Although some vaccination against NDV is carried out in the commercial poultry sector in Guyana, further studies are needed to determine the country’s NDV disease status. Suriname has reported the continued presence of NDV in poultry in consecutive reports to the OIE [13], but details on the disease occurrences are lacking. Belize reported an outbreak of ND in 2008 in commercial poultry, where mortality rates of 10.8% and 3% were recorded in domestic chicken and turkey flocks, respectively [33]. The Belize NDV isolates showed high levels of similarity to a 2007 NDV isolate from Honduras, all of which clustered into a new clade appearing to have evolved separately from other strains circulating in the Americas region [33]. Since the 2008 epidemic in Belize, outbreaks of NDV have continued to be reported, but only in backyard chicken in the southern districts of the country. The Belize Poultry Association, the Belize Agricultural Health Authority, the Ministry of Agriculture, and the Ministry of Health have partnered to execute a Newcastle disease community vaccination program in the affected districts of the country. Preliminary assessments show that there has been a high uptake of vaccination from communities, especially those that have reported ND outbreaks.

In the Caribbean island states, a severe outbreak of ND was reported in the DR in 2008. The outbreak was caused by a virulent strain of NDV with high genotypic divergence from known and ancestral strains; thus, the strain is considered a novel genotype [34]. Both the virus isolates from Belize and the DR were found to be virulent NDV strains based on intracerebral pathogenicity index (ICPI) scores, clinicopathological assessments, fusion protein cleavage site analysis, and genomic characterization. In the remaining Caribbean island states, there have only been sporadic reports of NDV being present and causing disease in poultry. The last reported occurrence of ND in Jamaica was in 1969, and no further reports of disease have emerged since then [35]. At the bottom of the island chain in T & T, an isolated outbreak of ND was reported to have occurred in Port of Spain in 1952, where birds exhibited classic ND clinical signs in a single flock of backyard chickens [36]. Characteristic severe clinical signs are, however, not always observed with NDV infection, as there are frequent reports of the circulation of low virulent strains of the virus that result in little or no clinical disease [37]. A recent serological study in T & T, where many of the larger poultry operations vaccinate their birds against NDV, reported the presence of NDV antibodies in unvaccinated layer birds, which had no previous history of clinical disease [20]. Seroprevalence levels were found to be 79.8% in unvaccinated layer birds from Trinidad and 80.5% in unvaccinated layer birds from Tobago. In 2014, an island wide survey of commercial poultry flocks in Grenada, where no NDV vaccination is carried out, reported that 47.7% of the tested birds had antibodies to NDV, but no history of clinical signs of disease were reported in the sampled birds [18]. As live NDV vaccines are commonly used globally, it is important to be able to differentiate between the circulation of low pathogenic field strains of NDV and circulating vaccine strains.

## 5. Infectious Laryngotracheitis Virus

ILTV, a member of the Herpesviridae family, is a highly contagious virus of poultry that can cause severe respiratory disease. The virus persists in infected birds for life [38]. Once the virus is present in a farm environment, it becomes extremely difficult to remove, due to its ability to become latent [39]. Because of this, vaccination against ILTV is not routinely carried out in the Caribbean, as the live ILTV vaccine brings with it the risk of introducing the virus into the region. Over the past 40 years, there have been multiple reports of ILTV outbreaks in various South American countries [40] but very few reports of outbreaks in the Caribbean region. There have been no reports of ILTV being present in poultry flocks in Belize, Guyana, or Suriname. Of the Caribbean island states, only T & T has reported the presence of ILTV. Trinidad experienced a confirmed isolated outbreak of infectious laryngotracheitis (ILT) in 2004 [35]. The means of introduction of ILTV onto the farm was never clearly determined. This outbreak was successfully controlled through the depopulation of all birds on the infected farm and other emergency response measures, such as disinfection and movement controls. To ensure that the virus was no longer present on the farm, the new birds introduced onto the farm were tested for the presence of antibodies to ILTV over their lifespan. A recent serological study carried out in layer birds on the islands of T & T revealed no antibodies against ILTV in Trinidad. In contrast, on the sister island of Tobago, 30.7% of the sampled layer birds were found to have ILTV antibodies, although no current or previous history of clinical signs were observed in birds on the antibody positive farms [20]. Since no ILTV vaccines have ever been used in T & T, it is possible that the circulating ILTV is a low virulent strain, or possibly a vaccine strain that has been introduced with imported birds. Further studies are needed to identify the genotype and phenotype of the ILTV circulating in the Tobago poultry to determine whether any serious disease risk is posed. Low virulent strains of ILTV have been reported to be circulating in the wider Latin American region and the virus has been reported to be present in nearby Brazil and Venezuela [40].

## 6. Avian Metapneumovirus

aMPV, a member of the virus family Paramyxoviridae, is an important respiratory virus of poultry, causing severe disease in turkeys, and to a lesser extent in chickens [41]. The virus emerged in the 1980s and is thought to have spread to various regions around the world through migratory birds [41]. The virus has been frequently reported as being present in the USA since the late 1990s [42], but there are few reports of virus circulation in the wider region. Although vaccines are available, they are not being used in poultry in the Caribbean region.

The only published reports of the presence of aMPV within the Caribbean region have emerged from Grenada and T & T. In a 2013 study, the authors reported aMPV antibodies to be present in 31.7% of broilers and 61.9% of layers from commercial flocks in Grenada [43]. In a recent serological study from T & T, high seroprevalence levels (67.5% in Trinidad and 100% in Tobago) were observed in layer birds [20]. Clinical signs were not reported in the antibody positive flocks in both studies and birds were not vaccinated against the virus. These results indicate that the virus is circulating in poultry in Grenada and T & T, posing a potential risk to commercial poultry, especially the turkey sector. Surveillance for aMPV by countries in the region, particularly those countries with large intensive poultry production, should be a priority, as serotypes of aMPV with increased virulence in chickens have been reported to be circulating in the USA [44]. This, coupled with the evidence of spread of aMPV throughout North America by wild and domestic birds [45], justifies the need for continued surveillance and monitoring of poultry within the Caribbean.

## 7. Infectious Bursal Disease Virus

IBDV, a member of the Birnaviridae family, causes a highly contagious disease of young chickens known as infectious bursal disease (IBD), also known as Gumboro disease. The disease is characterized by immunosuppression and mortality in 3- to 6-week-old broilers. The virus weakens the immune system leaving birds highly susceptible to secondary infections [46]. This virus is widely vaccinated against in the larger Caribbean countries with established intensive poultry industries. Very virulent strains of IBDV (vvIBDV) have been reported to be circulating in South American countries, causing disease in vaccinated birds [47,48,49]

Although IBDV has a worldwide distribution, there have been very few reports of the virus being present in the Caribbean region. There are no reports of IBDV circulation in Guyana, but there are some reports of detection of IDBV from Suriname and Belize [13]. Belize has experienced major outbreaks of IBDV in broiler flocks, which has resulted in the introduction of vaccination programs for broilers, as well as for parent breeder flocks, across the country. The vaccination programs, which include the regular monitoring of vaccine titers, have resulted in effective control of the disease in Belize.

In the Caribbean island states, Gumboro disease was first reported in Cuba in 1982 [50]. In 2012, an extensive 13-year retrospective report on the presence of IBDV in Cuba highlighted a series of outbreaks and changes in biosecurity measures within the country [51]. During the period from 1996–2002, 620 outbreaks were reported, with 120 separate outbreaks being reported in the year 2000. In 1996 alone, 245,921 poultry deaths by IBDV were reported in Cuba. These high levels of outbreaks being reported were thought to be due to a change in the vaccination program in Cuba. Poor vaccine dosage administration and issues with cold storage of the vaccines were thought to be the main causes for the apparent vaccine failure [51]. IBDV has also been confirmed to be circulating in the DR. The DR virus was further characterized and was reported to possess genetic characteristics consistent with vvIBDV [52]. A recent serological study carried out in T & T revealed very high seroprevalence levels (94.9% in Trinidad and 97.1% in Tobago) of antibodies to IBDV in unvaccinated layer birds [20]. On the island of Grenada, where no vaccination against IBDV is carried out, 40.35% of layer birds that were sampled had antibodies for IBDV [18]. These results from T & T and Grenada indicate that the virus is circulating, but the lack of clinical signs observed in the seropositive birds indicates either that low virulent strains of IBDV are circulating or that vaccine-derived strains are circulating. Further genetic analysis of the circulating field strains is needed to address this question.

## 8. Avian Adenoviruses

Multiple adenovirus species infect and cause disease in birds. Two of the most significant adenoviruses affecting poultry are fowl adenovirus group 1 (FADV Gp1) and egg drop syndrome virus (EDSV). Twelve serotypes of FADV Gp1 are known to exist, and several of these serotypes (serotypes 4, 7, 8, and 11) are responsible for causing serious disease in poultry, as well as significant economic losses to poultry industries worldwide [53]. Vaccines against specific serotypes of FADV Gp1 are available, but are not widely used in the Caribbean. In response to a serious outbreak of disease caused by FADVGp1 in 2005/2006, Guyana enacted legislation in July 2007 requiring that broiler chicks be vaccinated against the virus prior to sale at hatcheries. In Trinidad, broiler farms vaccinate occasionally in response to IBH outbreaks to prevent the significant economic losses that can follow.

For FADV Gp1, two distinct clinical conditions exist, the first known as inclusion body hepatitis (IBH), causes characteristic pale livers with pinpoint hemorrhages. The second condition, caused primarily by infection with serotype 4, is known as hydropericardium syndrome (HPS). Disease is characterized by an accumulation of fluid in the pericardial sac and results in higher levels of mortality than for IBH [54]. FADV Gp1 is found widely in both sick and healthy chickens and, like IBDV, it suppresses the immune system making infected birds susceptible to other infections [55]. EDSV is an avian adenovirus from Group 3. It causes a drop in egg production, as well as the production of defective eggs [56,57]. These clinical signs, though not exclusive to EDSV [41,58], can lead to severe economic losses for poultry farmers.

Avian adenoviruses are not widely reported to be present in the Caribbean region, but they are present and circulating in the Americas [56,59,60]. No known reports of disease caused by avian adenoviruses have been reported from the mainland states of Belize and Suriname. In Guyana, though reporting is limited, the virus is known to have caused severe outbreaks of disease in poultry along the East Bank of Demerara and the Soesdyke Linden highway from 2005–2006. By early 2007, the virus was causing outbreaks of disease further south and east in Berbice, which resulted in the implementation of legislation (Regulation 13 of 2007) requiring that broiler chicks be vaccinated prior to sale at hatcheries. Cases of IBH have been reported in Cuba, where in 1983 the disease was diagnosed, associated with the presence of IBDV, in broilers [61]. A mixed infection of both viruses was confirmed, along with histological changes in both the liver and the Bursa of Fabricius in 28-day-old broilers. In a recent serological survey carried out on the islands of T & T, 100% of the layer birds sampled on both islands tested antibody positive for FADV Gp1 viruses. In contrast, 67% and 100% of the sampled birds were antibody positive for EDSV on the islands of Trinidad and Tobago, respectively [20]. These results reveal that avian adenoviruses are circulating in T & T, but they are not necessarily causing overt clinical disease in infected birds. They may, however, be suppressing the immune system of infected birds, making them more susceptible to other potential pathogens.

## 9. Conclusions

Food security, as well as economic growth of the agricultural sectors in many Caribbean countries, is heavily dependent on poultry production, which in turn is dependent on the health status of the farmed birds. This review focuses on serological, molecular, and phylogenetic studies related to eight high-priority economically important viral pathogens of poultry in Caribbean island states, as well as in the Caribbean Community (CARICOM) mainland member states, Belize, Guyana, and Suriname. The review reveals evidence for sporadic outbreaks of respiratory disease caused by notifiable viral pathogens (AIV, IBV, NDV, and ILTV), as well as outbreaks of diseases caused by immunosuppressive viral pathogens (IBDV and FADV Gp1) across Caribbean countries.

This review highlights knowledge gaps from a pathogen and a country perspective for targeting surveillance activities in the region and highlights the absence of reported data from some Caribbean countries. It is not, however, known whether this lack of data is due to a reluctance to report the presence of disease, a lack of local diagnostic capacity and capability within countries, or simply the fact that no disease is present in the country to report. Although active surveillance in response to outbreaks of disease is carried out in many of the larger poultry-producing countries of the Caribbean, limited levels of passive surveillance is carried out in most Caribbean states. Many of the smaller Caribbean countries have limited or no laboratory capacity, making it difficult or impossible to carry out laboratory confirmation of diseases-causing agent in-country. In the larger broiler-producing countries of the Caribbean, the privately owned broiler producers often hire their own private vets and then send samples for disease investigations to private labs in the USA. The results from these investigations are not always published, so it is possible that some information related to disease outbreaks in these countries are not represented in this review.

Undoubtedly, challenges exist in many Caribbean countries related to gaining funding to carry out regular active and passive surveillance for avian diseases and to enabling the rapid and efficient detection and diagnosis of avian pathogens in-country. Funding and training is required to enable national diagnostic laboratories to carry out rapid, accurate diagnosis, as well as molecular identification and characterization of circulating avian viruses. Improved levels of surveillance, diagnosis and research is clearly required throughout the region in order to improve knowledge related to the presence and circulation of avian viruses. Knowing this information would enable the improvement of current control and prevention strategies in relation to viral diseases of poultry across the Caribbean region.
